# Micro-RNA Feedback Loops Modulating the Calcineurin/NFAT Signaling Pathway

**DOI:** 10.3390/ncrna2020003

**Published:** 2016-05-20

**Authors:** Shichina Kannambath

**Affiliations:** Infection and Immunity, St. George’s University of London, Cranmer Terrace, London SW17 0RE, UK; skannamb@sgul.ac.uk; Tel.: +44-20-8725-2676

**Keywords:** miRNA targets, calcineurin/NFAT signaling pathway, pathway regulation, feedback loops

## Abstract

Nuclear factor of activated T cells (NFAT) is a family of transcription factors important for innate and adaptive immune responses. NFAT activation is tightly regulated through the calcineurin/NFAT signaling pathway. There is increasing evidence on non-coding RNAs such as miRNAs playing a crucial role in regulating transcription factors and signaling pathways. However, not much is known about microRNAs (miRNAs) targeting the calcineurin/NFAT signaling pathway involved in immune response in human. In this study, a comprehensive pathway level analysis has been carried out to identify miRNAs regulating the calcineurin/NFAT signaling pathway. Firstly, by incorporating experimental data and computational predictions, 191 unique miRNAs were identified to be targeting the calcineurin/NFAT signaling pathway in humans. Secondly, combining miRNA expression data from activated T cells and computational predictions, 32 miRNAs were observed to be induced by NFAT transcription factors. Finally, 11 miRNAs were identified to be involved in a feedback loop to modulate the calcineurin/NFAT signaling pathway activity. This data demonstrate the potential role of miRNAs as regulators of the calcineurin/NFAT signaling pathway. The present study thus emphasizes the importance of pathway level analysis to identify miRNAs and understands their role in modulating signaling pathways and transcription factor activity.

## 1. Introduction

Nuclear factor of activated T cells (NFAT) is a family of transcription factors that was first described as a part of a protein complex, which altered transcription of the interleukin-2 (IL-2) gene. The NFAT family consists of five members: NFATc1 (NFAT2), NFATc2 (NFAT1), NFATc3 (NFAT4), NFATc4 (NFAT3) and NFAT5, in which the first four NFATs are regulated by calcium signaling [1,2]. NFAT transcription factor activities are tightly regulated through the calcineurin/NFAT signaling pathway. This pathway is an essential player in immune responses including cytokines’ production of TNF-α and IL-10 [1]. When T cells are activated, the intracellular calcium level increases and binds to a protein called calmodulin. The calcium-bound calmodulin interacts with calcineurin, which in turn dephosphorylates NFATs into its active form. Dephosphorylated NFATs translocate from the cytoplasm to the nucleus and induce NFAT-dependent transcription [2,3]. The calcineurin/NFAT signaling pathway is thus crucial for the adaptive immune response and T cell activation [4]. Together with its role in adaptive immunity, NFATs are also involved in innate immune responses against fungal and bacterial infection [5,6].

MicroRNAs (miRNAs) are short (18–22 nucleotides in length), endogenous, non-coding single-stranded RNAs that regulate expression of target mRNAs. miRNAs have emerged as one of the key regulators of gene expression [7]. Nearly one-third of the genes in the human genome are estimated to be regulated by the ~2000 mature miRNAs so far identified [8]. miRNAs can activate or inhibit the function of a signaling pathway by translational inhibition of the component genes [9,10].

There is growing evidence on miRNA regulating the calcineurin/NFAT signaling and its involvement in innate and adaptive immune responses [11,12,13,14]. For example, NFATc2 in CD4 T cells was shown to be regulated by miR-184 and miR-568 was shown to inhibit the activation of T cells by targeting NFAT5 [15,16]. Also, miRNA-124 has been shown to repress activation of the NFATs that lead to an altered immune response [11]. Another recent study demonstrated that overexpression of miR-9 in T cells activates NFAT by repressing Dyrk1, a kinase that phosphorylates NFAT [17]. A few miRNAs, such as miR-23a, have been reported to be controlled by NFAT transcription factor [18]. Apart from the immune cells, a number of studies have explored miRNA regulation on the calcineurin/NFAT signaling pathway in cardiac hypertrophy [19,20,21,22,23,24]. NFATc4 was targeted by miR-133 in cardiac hypertrophy [20] and miR-199b has been shown to regulate the calcineurin/NFAT signaling pathway by targeting Dyrk1A [25]. Similarly, NFAT was shown to interact with miR-25 to regulate the functional activity of NFAT [24]. MiR19a/b was studied to positively regulate the NFAT activation and this effect was observed to be suppressed with calcineurin inhibitors [23]. Though there are a few studies suggesting miRNAs regulate the calcineurin/NFAT signaling pathway, a comprehensive study to identify all miRNAs targeting this pathway and their effect on pathway regulation is lacking and has not been explored in detail. In addition, not much is known about the miRNAs induced by NFAT transcription factors. Only a few studies have experimentally explored miRNA function in the context of network regulation [21]. Emerging studies have shown the role of miRNAs in such feedback loops to regulate signaling pathways and transcription factor activities [26]. However, miRNAs involved in a feedback loop to modulate the calcineurin/NFAT signaling pathway activity are unknown. Thus, a comprehensive systems-level analysis is required to understand the role of miRNAs and the feedback loops involving these miRNAs to regulate the calcineurin/NFAT pathway.

A number of computational methods have been developed to predict the miRNA targets based on sequence and structural information of miRNA-mRNA interaction [27,28,29,30,31]. In addition, there are high-throughput sequencing–based experimental studies exploring the expression patterns of miRNAs in human and mouse [12,32]. In this study, computational prediction methods were combined with published experimental data for a precise prediction of miRNAs involved in modulating the activities of the calcineurin/NFAT signaling pathway. In addition, miRNAs targeting members of the pathway and miRNAs induced by NFAT transcription factor were identified. Further, miRNAs that are potentially involved in a feedback loop that would regulate the calcineurin/NFAT signaling pathway in humans were identified through a comprehensive systems-level analysis.

## 2. Results

### 2.1. miRNAs Targeting the Calcineurin/NFAT Signaling Pathway

There are a few studies describing the role of miRNAs in regulating the calcineurin/NFAT signaling pathway [11,15,16,17,18,20]. In this study, combining computational prediction algorithms and incorporating experimental data, miRNAs targeting the calcineurin/NFAT signaling pathway were identified. To undertake this analysis, 23 key genes, NAFT, Protein Phosphatase 3, Catalytic Subunit, Alpha Isozyme (PPP3CA), Catalytic Subunit, Beta Isozyme, (PPP3CB), Protein Phosphatase 3, Regulatory Subunit B, Alpha (PPP3R1), Protein Phosphatase 3, Regulatory Subunit B, Beta (PPP3R2), Regulator Of Calcineurin 1 (RCAN1), Glycogen synthase kinase 3 beta (GSK3B), Dual specificity tyrosine-phosphorylation-regulated kinase 1A (DYRK1A), Homer Scaffolding Protein 2 ( HOMER2), Inositol 1,4,5-Trisphosphate Receptor Type 1 (ITPR1), Mitogen-Activated ProteinKinase Kinase Kinase 7 ( MAP3K7), TAK1-binding protein (TAB), Casein Kinase (CSNK1A1/CK1), Leucine-Rich Repeat Kinase 2 (LRRK2), Calcineurin-Binding Protein 1 (CABIN1), A-Kinase Anchor Protein 5 (AKAP5), calmodulin 1 (CALM1), Stromal Interaction Molecule 1 (STIM1) and Orai Calcium Release-Activated Calcium Modulator 1 (ORAI1), from the calcineurin/NFAT signaling pathway described to be important for regulating NFAT activity were selected based on the literature review (Table 1) [33,34,35,36,37,38]. A combination of six target prediction algorithms, StarBase [39,40], TargetScan [41,42], miRDB [43,44], TarBase 7 [45,46], DIANA [47,48] and miRNAMap [49,50], was used to identify miRNAs targeting the selected genes regulating NFAT activation. Most of these algorithms incorporate experimental validation of miRNA expression data. Since these algorithms are based on different prediction methods, sequences and/or structural interaction between miRNA and mRNA, the prediction overlap of these algorithms remains very low. However, combined, prediction algorithms are shown to give better a prediction of the targets [51]. These algorithms incorporate features that include sequence composition, secondary structure and species conservation to predict miRNA targets. The miRNA targets predicted by all six algorithms were used for further analysis. The computational analysis predicted 4679 combinations of mRNA-miRNA interactions involving 1961 unique miRNAs targeting the calcineurin/NFAT signaling pathway (Supplementary data).

The miRNA targeting the calcineurin/NFAT signaling pathway was further refined by incorporating experimental data on miRNA target validation. Experimental validation data was collected from the miRTarBase database [28,62]. A subset of 230 miRNAs with experimentally validated data on targeting the calcineurin/NFAT signaling pathway was selected for subsequent analysis (Supplementary data). This miRNA subset was further refined by incorporating miRNA-expressed data from human immune cells (Supplementary data). Since the calcineurin/NFAT signaling pathway is known to be important for regulating the T cell development and immune response [4], miRNAs expressed in immune cells were collected from miRmineHuman miRNA expression (http://guanlab.ccmb.med.umich.edu/mirmine/index.html) database [63]. By incorporating this expression data, 191 miRNAs targeting the calcineurin/NFAT signaling pathway were identified (Supplementary data). The negative regulators of this pathway, Glycogen synthase kinase 3 beta (GSK3B), Dyrk1A, CSNK1A1 and RCAN1, were among the highly targeted genes, with 26, 14, 14 and 11 miRNA targets, respectively. The positive regulators of this pathway, PPP3R1, MAP3K7, TAB2, CALM1 and ITPR1, were also observed to be targeted by 19, 14, 14, 15 and 12 miRNAs, respectively (Supplementary data).

### 2.2. miRNAs Induced by NFAT Transcription Factor Family

miRNAs are known to be transcribed mainly in two different ways: (a) as part of an mRNA or (b) individually with a separate promoter [64]. The majority of the miRNAs are thought to form an independent transcription unit and the rest are transcribed as part of the annotated genes [65]. However, it is not clearly understood which miRNAs are transcribed as part of an mRNA or have independent transcription. Not many studies are done on transcription factors that transcribe these miRNAs. However, a number of computational prediction methods have been developed to predict transcription factors that can bind to the miRNA promoter region and potentially transcribe it [66]. There are ChIP-seq experimental data available on transcription factors inducing miRNA expression in ChIPBase (http://deepbase.sysu.edu.cn/chipbase/index.php) [67,68]. However, miRNAs induced by the NFAT family of transcription factors are not available from the above study.

In the current study, miRNAs induced by the NFAT transcription factors were predicted by combining computational predictions and published experimental data. To conduct this analysis, miRNA sequence coordinates were collected from the miRBase database [8]. Promoter regions of the miRNAs were obtained through the UCSC genome browser [69,70] and promoter sequences for 1443 human miRNAs were collected from UCSC. The NFAT binding sites on these promoter regions were predicted using transcription factor prediction algorithms TFSEARCH (http://diyhpl.us/~bryan/irc/protocol-online/protocol-cache/TFSEARCH.htmls) [71] and PROMO (http://alggen.lsi.upc.es/cgi-bin/promo_v3/promo/promoinit.cgi?dirDB=TF_8.3) [66,72]. Prediction results from these algorithms were combined to identify 536 miRNAs with an NFAT transcription factor binding site on the promoter region (Supplementary data).

**Figure 1:**
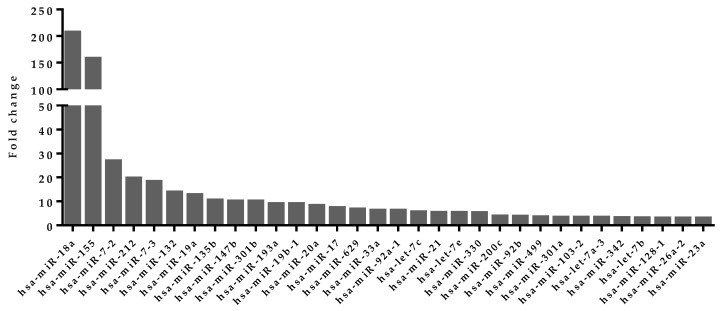
List of 32 miRNAs potentially induced by NFAT transcription factor with the fold change of expression in activated T cells. These miRNAs were observed to be highly overexpressed in activated T cells compared to non-activated T cells.

This computational prediction of the miRNAs induced by NFAT transcription factors was further refined by incorporating published miRNA expression data in activated and non-activated T cells in humans [32]. In an activated T cell, NFAT translocate into the nucleus and starts transcribing genes under its control. Thus, it was hypothesized that the miRNAs induced by NFATs would get transcribed in activated T cells and would get upregulated in activated T cells compared to non-activated T cells. Based on this hypothesis, miRNA expression data for human T cells were further analyzed. Ninety-five miRNAs upregulated by 1.5-fold or more in active T cells were selected (Supplementary data). A subset of these miRNAs was observed to have an NFAT transcription factor binding site on their promoter region. Thirty-two miRNAs from this subset that were identified to have an NFAT binding region and to be upregulated in activated T cells were selected, because they are potentially induced by NFAT transcription factor (Figure 1).

### 2.3. miRNAs in Negative or Positive Feedback Loop to Modulate the Calcineurin/NFAT Signaling Pathway Activity

**Figure 2:**
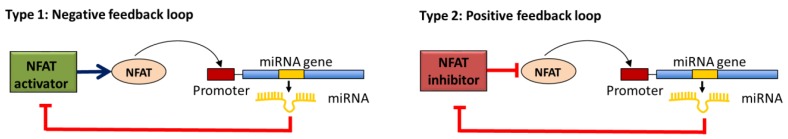
Schematic representation of feedback loops regulating the calcineurin/NFAT signaling pathway. In a negative feedback loop (type 1), miRNAs transcribed by NFAT inhibit NFAT activation, whereas in a positive feedback loop (type 2), miRNAs transcribed by NFAT repress the expression of the NFAT inhibitor which results in the activation of NFAT.

Signaling pathways such as the calcineurin/NFAT pathway are tightly regulated through positive or negative feedback loops to reduce fluctuations in gene expression [73,74,75]. Previous studies have shown the role of miRNAs in such feedback loops [26]. While miRNAs operate through a repressive mechanism, their function in signaling pathways can result in activation or repression of the pathways. Not much is known about the miRNAs involved in a feedback loop to regulate the calcineurin/NFAT signaling pathway. An miRNA feedback loop can be of two types: a negative feedback loop, where miRNAs transcribed by NFAT transcription factor inhibit NFAT activation (translocation to nucleus) by repressing NFAT or NFAT activators (type 1), and a positive feedback loop, where miRNAs transcribed by NFAT transcription factors repress the expression of the NFAT inhibitor, which results in the activation of NFAT (type 2) (Figure 2).

In this study, by combining miRNAs targeting the calcineurin/NFAT signaling pathway and miRNAs induced by NFAT transcription factor, miRNAs involved in feedback loops to modulate the calcineurin/NFAT signaling pathway were identified. Computational predictions combined with experimentally validated data have identified 191 miRNAs targeting the calcineurin/NFAT signaling pathway and 32 miRNAs induced by NFAT transcription factors. By combining these two sets of miRNAs, a subset of 11 miRNAs (Table 2) were observed to be potentially involved in a feedback loop to regulate the calcineurin/NFAT signaling pathway. Out of the 11 miRNAs, six (hsa-miR-21-3p, hsa-let-7b-5p, hsa-miR-17-5p, hsa-miR-19a-3p, hsa-miR-92b-3p and hsa-miR-17-3p) are involved in a negative feedback loops that modulates NFAT activity down and five miRNAs (hsa-miR-21-5p, hsa-miR-181c-5p, hsa-let-7c-5p, hsa-let-7b-3p and hsa-miR-155-5p) are involved in a positive feedback loops facilitating NFAT activation.

## 3. Discussion

**Figure 3:**
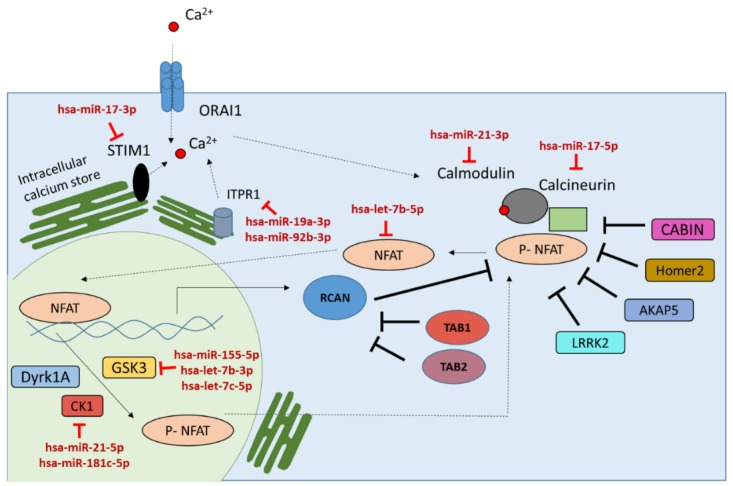
Schematic representation of the calcineurin/NFAT signaling pathway and the miRNAs involved in feedback loops to modulate the pathway activities. All the selected 23 genes associated with the calcineurin/NFAT signaling pathway in humans have miRNAs targeting those genes which are involved in feedback loops to regulate the calcineurin/NFAT signaling pathway.

The calcineurin/NFAT signaling pathway is an important immune regulatory pathway in vertebrates. This pathway regulates the activation of a family of NFAT transcription factors, which upon activation regulate the gene expression of important immune regulatory cytokines [1]. Over the past decade, the importance of miRNAs in the regulation of signaling pathways, such as the calcineurin/NFAT signaling pathway, has become more and more evident [76]. miRNAs through their repressive function can modulate diverse signal transduction pathways [9]. However, apart from a few miRNAs, not much is known about the miRNAs involved in regulating the calcineurin/NFAT signaling pathway [11,15,17]. In the present study, computational predictions, experimental validation and miRNA expression data were combined to identify miRNA feedback loops regulating the calcineurin/NFAT signaling pathway. The concept of feedback regulation of pathways has been known for over 100 years [77]. A variety of studies in the literature have identified positive or negative feedback loops in the calcineurin/NFAT signaling pathway [1,25,35,53]. However, miRNA feedback loops are much less studied, and currently no information is available on miRNA feedback loops regulating the calcineurin/NFAT signaling pathway. The present study describes 11 miRNAs (Table 2) potentially involved in feedback loops to regulate the calcineurin/NFAT signaling pathway. These miRNAs were systematically identified based on their involvement in targeting the calcineurin/NFAT pathway and on computational predictions to be a potential target of NFAT transcription factor. miRNAs observed to be involved in negative feedback loops, hsa-miR-21-3p, hsa-let-7b-5p, hsa-miR-17-5p, hsa-miR-19a-3p, hsa-miR-92b-3p and hsa-miR-17-3p, are targeting the NFAT activators, thus modulating down the NFAT activity. miRNAs observed to be part of the positive feedback loops, hsa-miR-21-5p, hsa-miR-181c-5p, hsa-let-7c-5p, hsa-let-7b-3p and hsa-miR-155-5p, target the negative regulators of the NFAT, thus facilitating NFAT activation (Figure 3).

The let-7 miRNAs, identified in this study to be involved in both negative and positive feedback loops, are part of the highly conserved miRNA families across different species. The let-7 family of proteins is described to be expressed in immune cells (miRmine database) and is overexpressed in T cells upon activation [32]. Various experimental validation studies have shown that the let-7 family of miRNAs targets members of the calcineurin/NFAT signaling pathway (hsa-let-7b-3p, hsa-let-7c-5p targeting GSK3 and hsa-let-7b-5p targeting NFATc1) [78,79]. Let-7f, one member of the let-7 miRNA family, has previously been shown to positively modulate NF-κB signaling by targeting an inhibitor of the pathway, A20 [76]. Thus, upregulation of let-7f is shown to be beneficial for the immune cells to control infection [76]. The current study demonstrates the potential role of hsa-let-7b-3p and hsa-let-7c-5p in a positive feedback loop by targeting the negative regulator of NFAT, GSK3. This data suggest, similar to the NF-κB signaling, the potential role of let-7 in activating the NFAT signaling pathway by targeting GSK3, which would be beneficial for immune response.

The next set of miRNAs observed to be involved in feedback loops, hsa-miR-17-3p, hsa-miR-17-5p, hsa-miR-19a-3p and hsa-miR-92b-3p, are part of the miR-17~92 cluster. In humans, the miR-17~92 cluster is composed of six members from four different seed families—miR-17, miR-18a, miR-19a, miR-20a, miR-19b-1, and miR-92-1. In mice, overexpression of the miR-17~92 cluster has been shown to be associated with an increased CD4+ T cell population and autoimmune disease [80]; however, similar data is not available for humans. Transcription factors c-Myc and E2F are shown to be associated with activation of the miR-17~92 cluster miRNAs. In addition, the miR-17~92 cluster is involved in a feedback loop regulating E2F family members [80]. The four members of the miR-17~92 cluster, hsa-miR-17-3p, hsa-miR-17-5p, hsa-miR-19a-3p and hsa-miR-92b-3p, from this study have been observed to be involved in a negative feedback loop to target the calcineurin/NFAT signaling pathway. Three of them are observed to be targeting the genes associated with calcium release (hsa-miR-17-3p targeting STIM1, hsa-miR-19a-3p and hsa-miR-92b-3p targeting ITPR1). This data suggests that members of the miR-17~92 cluster are potentially involved in negatively regulating the calcineurin/NFAT signaling pathway, potentially to reduce over-activation of the immune response.

Bazzoni *et al.* have demonstrated the miRNA-mRNA interaction between hsa-miR-155-5p and GSK3, and explored the potential involvement of hsa-miR-155 in the regulation of innate immune response [81]. There are several studies exploring the role of hsa-miR-155 in regulating immune responses, and in mice miR-155 has been shown to be crucial for T cell differentiation and proliferation [80]. In humans, miR-155 expression was shown to be associated with transcription factor NF-κB; however, in mice, miR-155 was shown to be activated via the NFAT pathway [81,82]. However, no study has yet explored the role of NFAT transcription factor as an inducer of hsa-miR-155 expression in human immune cells. In this study, the transcription factor binding region analysis identified a potential NFAT binding site on the hsa-mir-155 promoter region and also demonstrated the role of hsa-miR-155 in positive feedback loops. Thus, similar to let-7, by targeting GSK3 hsa-miR-155 potentially activates the calcineurin/NFAT pathway, which is important for T cell proliferation.

Similar to miR-155, hsa-miR-21 has also been shown to be a major regulator of Th1 *versus* Th2 T cell response [83]. In this study, hsa-miR-21-5p has been shown to target CK1 (CSNK1A1) and to be involved in a positive feedback loop activating the calcineurin/NFAT signaling pathway. A microarray study done by Gabriely *et al.* has validated the miRNA-mRNA interaction between hsa-miR-21-5p and CK1 (CSNK1A1) [84]. Being involved in a positive feedback loop and by targeting CK1, miR-21 might be contributing to T cell proliferation through activating the NFAT transcription factor. Interestingly, hsa-miR-21-3p has been observed to be targeting the CALM1 gene and to be involved in a negative feedback loop. It is known that miRNAs from the same pre-miRNA could have different functional properties depending on the differences in the seed sequence or nucleotides at the 5′ and 3′ ends [85]. The second miRNA target of CSNK1A1, hsa-miR-181c, however, has not been studied in detail for its function in T cell activation and immune responses. hsa-miR-181c is part of the miR-181 family, consisting of miR-181a, miR-181b and miR-181c. Kishore *et al.*, using the cross-linking and immunoprecipitation (CLIP) method, have validated the interaction between CSNK1A1 and hsa-miR-181c [86]. In the present study, hsa-miR-181c has been described to be potentially involved in a positive feedback loop to activate the calcineurin/NFAT pathway by silencing the negative of the pathway, CSNK1A1. These data thus suggest a potential role of hsa-miR-181c in silencing CSNK1A1, which would activate the NFAT pathway and immune response.

Taken together, feedback loops involving miRNAs demonstrate the crucial role of miRNAs in modulating the calcineurin/NFAT signaling pathway. Additional comprehensive pathway level studies including experimental validations need to be carried out to identify these miRNAs and understand their role in signaling pathways.

## 4. Materials and Methods

### 4.1. Computational Prediction of miRNA Targeting Members of the Calcineurin/NFAT Pathway

Six miRNA target prediction algorithms, StarBase (v2.0 release at September 2013) (http://starbase.sysu.edu.cn/index.php/) [40], TargetScan (Release 7.0, August 2015) (http://www.targetscan.org/) [42], DIANA (2015) (http://diana.imis.athena-innovation.gr/DianaTools/index.php) [48], miRDB (2015) (http://mirdb.org/miRDB/index.html) [44], miRNAMap (http://mirnamap.mbc.nctu.edu.tw/index.php) [50] and TarBase 7 (v7.0,2015) (http://diana.imis.athena-innovation.gr/DianaTools/index.php?r=tarbase/index) [46] were used for computational prediction of miRNAs targeting 23 members of the calcineurin/NFAT pathway. The algorithms were used using their default parameters and miRNAs predicted by at least one of the prediction algorithms were selected for subsequent analysis. The computational prediction identified 4679 combinations of mRNA-miRNA interactions with 1961 unique miRNAs targeting the calcineurin/NFAT signaling pathway (Supplementary data).

### 4.2. Experimental Validation Data on miRNA Targets

miRNA target validation data was collected from the database miRTarBase (http://mirtarbase.mbc.nctu.edu.tw/index.php) [62]. List of all miRNAs targeting the 23 members of the calcineurin/NFAT pathway were collected. Experimental validation data on 840 miRNA-mRNA interactions were collected from miRTarBase (Supplementary data).

### 4.3. miRNA Expressed in PBMCs

miRNA expression data was collected from miRmine—Human miRNA expression database (http://guanlab.ccmb.med.umich.edu/mirmine/index.html) [63]. A subset of 692 miRNAs were collected which were expressed in human PBMCs with reads per million (RPM) >20 (Supplementary data).

### 4.4. miRNA Promoter Sequences and NFAT Transcription Factor Binding Site

Genomic coordinates of the miRNA precursor were obtained from miRBase database (Release 20: June 2013). The miRNA promoter sequences (5000 nt) for human (h19) was downloaded from UCSC Genome Browser website (http://genome.ucsc.edu/index.html) [70]. The NFAT transcription factor binding sites on the miRNA promoter regions were retrieved using two transcription factor binding site prediction algorithms: TFSEARCH (http://diyhpl.us/~bryan/irc/protocol-online/protocol-cache/TFSEARCH.htmls) [71] and PROMO (http://alggen.lsi.upc.es/cgi-bin/promo_v3/promo/promoinit.cgi?dirDB=TF_8.3) [66,72]. (Supplementary data).

### 4.5. Upregulated miRNA Selection from Activated Human T Cells

Expression profile of miRNAs from activated or non-activated T cells for human was collected from previously published datasets [32]. miRNAs were sorted based on their expression fold change in activated T cells compared to the non-activated cells. Seventy-three miRNAs with fold change >1.5 were selected for further analysis (Supplementary data).

## 5. Conclusions

A combination of computational prediction algorithms and experimental data available on miRNA expression and target predictions was used to identify possible miRNA feedback loops modulating the calcineurin/NFAT signaling pathway in humans. The feedback loop mechanism described in this study highlights the central role of miRNAs in regulating NFAT activity. Further studies need to be undertaken, especially experimental validation, which will lead to novel insights into the nature of miRNA modulation of the calcineurin/NFAT signaling pathway. Overall, this study demonstrates the importance of a comprehensive systems-level analysis in identifying the indirect role of miRNAs in activating or silencing pathway response, such as that of the calcineurin/NFAT signaling pathway.

## Figures and Tables

**Table 1:** Members of the calcineurin/NFAT signaling pathway analyzed in this study. Twenty-three key members of the calcineurin/NFAT signaling pathway were selected based on literature survey.

Gene	Description	
NFATc1	Family of transcription factors that are expressed in the vertebrates. These transcription factors induce expression of cytokine genes important for immune response.	[38,52]
NFATc2		
NFATc3		
NFATc4		
PPP3CA	Calcium-dependent, calmodulin-stimulated protein phosphatase. It dephosphorylates the NFAT transcription factors and facilitates nuclear transport of NFAT.	[33]
PPP3CB		
PPP3R1		
PPP3R2		
RCAN1	Transcribed by NFAT transcription factor and interacts with calcineurin to inhibit calcineurin-dependent signaling pathways.	[38,53]
GSK3B	Phosphorylates NFAT into its inactive form and facilitates the cytoplasmic transport of NFAT.	[38,54,55]
DYRK1A		
HOMER2	Is a negative regulator of T cell activation and binds with NFAT to compete with calcineurin binding.	[34]
ITPR1	Intracellular channel that mediates calcium release from the endoplasmic reticulum.	[38,56]
MAP3K7	Selectively induces calcineurin-NFAT signaling through direct phosphorylation of RCAN1.	[35]
TAB1	Phosphorylated RCAN1 and inhibits RCAN1 from binding to calcineurin.	[35]
TAB2		
CSNK1A1/CK1	Works as a negative inhibitor of the NFAT by phosphorylation and mediates its cytoplasmic translocation.	[57]
LRRK2	Negative regulator of the transcription factor NFAT and is a component of a complex that includes the large non-coding RNA NRON (an NFAT repressor).	[58]
CABIN1	Binds specifically to the activated form of calcineurin and inhibits calcineurin-mediated signal transduction and NFAT nuclear translocation.	[36]
AKAP5	Inhibits calcineurin-dependent dephosphorylation of NFAT	[59]
CALM1	Calmodulin is a calcium binding protein that activates the calcineurin.	[37]
STIM1	Ca2+ sensor proteins on the endoplasmic reticulum, which is an indispensable part in the activation of store-operated Ca2+ channels (SOC).	[60]
ORAI1	Calcium channel subunit that is activated by the calcium sensor STIM1 when calcium stores are depleted.	[61]

**Table 2:** List of miRNAs potentially involved in a feedback loop to modulate the calcineurin/NFAT signaling pathway. Eleven miRNAs are identified in this study to be involved in a feedback loop with target genes and expression in human activated T cells.

miRNAs Involved in Feedback Loops	Target Genes	miRNA Average Expression in Activated T Cells (RPM)
hsa-miR-21-3p	CALM1	191
hsa-let-7b-5p	NFATC1	8617
hsa-miR-17-5p	PPP3R1	778
hsa-miR-17-3p	STIM1	859
hsa-miR-19a-3p	ITPR1	4379
hsa-miR-92b-3p	ITPR1	254
hsa-miR-21-5p	CSNK1A1	372,670
hsa-miR-181c-5p	CSNK1A1	321
hsa-let-7c-5p	GSK3A	889
hsa-let-7b-3p	GSK3B	77
hsa-miR-155-5p	GSK3B	282,932

## Supplementary Materials

The following are available online at www.mdpi.com/2311-553X/2/2/3/s1, Supplementary data.

## Abbreviations

The following abbreviations are used in this manuscript:

NFAT	Nuclear factor of activated T cells
PPP3CA	Calmodulin-Dependent Calcineurin A Subunit Alpha Isoform
RCAN	RCAN Family Member/ Down Syndrome Candidate Region 1-Like Protein
GSK	Glycogen Synthase Kinase
DYRK1A	Dual-Specificity Tyrosine-(Y)-Phosphorylation Regulated Kinase 1A
HOMER2	Homer Scaffolding Protein
ITPR1	Inositol 1,4,5-Trisphosphate Receptor, Type 1
MAP3K7	Mitogen-Activated Protein Kinase Kinase Kinase 7
TAB	TGF-Beta Activated Kinase 1/MAP3K7 Binding Protein
CSNK1A1	Casein Kinase 1, Alpha 1
LRRK	Lrrk Leucine-rich repeat kinase
CABIN	Calcineurin Binding Protein
AKAP	A-kinase anchor proteins
CALM	Calmodulin
STIM	stromal interaction molecule
ORAI	Calcium Release-Activated Calcium Modulator
miRNA	micro RNA
RPM	Reads Per Million

## References

[1] Wu H., Peisley A., Graef I.A., Crabtree G.R. (2007). NFAT signaling and the invention of vertebrates. Trends Cell Biol..

[2] Rao A., Luo C., Hogan P.G. (1997). Transcription factors of the NFAT family: Regulation and function. Annu. Rev. Immunol..

[3] Hogan P.G., Chen L., Nardone J., Rao A. (2003). Transcriptional regulation by calcium, calcineurin, and NFAT. Genes Dev..

[4] Macian F. (2005). NFAT proteins: Key regulators of T-cell development and function. Nat. Rev. Immunol..

[5] Fric J., Zelante T., Wong A.Y.W., Mertes A., Yu H.-B., Ricciardi-Castagnoli P. (2012). NFAT control of innate immunity. Blood.

[6] Fric J., Lim C.X.F., Koh E.G.L., Hofmann B., Chen J., Tay H.S., Mohammad Isa S.A.B., Mortellaro A., Ruedl C., Ricciardi-Castagnoli P. (2012). Calcineurin/NFAT signalling inhibits myeloid haematopoiesis. EMBO Mol. Med..

[7] Bartel D.P. (2009). Review MicroRNAs : Target Recognition and Regulatory Functions. Cell.

[8] Kozomara A., Griffiths-Jones S. (2011). miRBase: Integrating microRNA annotation and deep-sequencing data. Nucleic Acids Res..

[9] Davis B.N., Hilyard A.C., Lagna G., Hata A. (2008). SMAD proteins control DROSHA-mediated microRNA maturation. Nature.

[10] Avraham R., Yarden Y. (2011). Feedback regulation of EGFR signalling: Decision making by early and delayed loops. Nat. Rev. Mol. Cell Biol..

[11] Kang K., Peng X., Zhang X., Wang Y., Zhang L., Gao L., Weng T., Zhang H., Ramchandran R., Raj J.U. (2013). MicroRNA-124 Suppresses the Transactivation of Nuclear Factor of Activated T Cells by Targeting Multiple Genes and Inhibits the Proliferation of Pulmonary Artery Smooth Muscle Cells. J. Biol. Chem..

[12] Kuchen S., Resch W., Yamane A., Kuo N., Li Z., Chakraborty T., Wei L., Laurence A., Yasuda T., Peng S. (2010). Regulation of microRNA expression and abundance during lymphopoiesis. Immunity.

[13] Ceppi M., Pereira P.M., Dunand-Sauthier I., Barras E., Reith W., Santos M.A., Pierre P. (2009). MicroRNA-155 modulates the interleukin-1 signaling pathway in activated human monocyte-derived dendritic cells. Proc. Natl. Acad. Sci. USA.

[14] Yang G., Yang L., Zhao Z., Wang J., Zhang X. (2012). Signature miRNAs Involved in the Innate Immunity of Invertebrates. PLoS ONE.

[15] Weitzel R.P., Lesniewski M.L., Haviernik P., Kadereit S., Leahy P., Greco N.J., Laughlin M.J. (2009). MicroRNA 184 regulates expression of NFAT1 in umbilical cord blood CD4 + T cells. Blood.

[16] Li W., Kong L.-B., Li J.-T., Guo Z.-Y., Xue Q., Yang T., Meng Y.-L., Jin B.-Q., Wen W.-H., Yang A.-G. (2013). MiR-568 inhibits the activation and function of CD4+ T cells and Treg cells by targeting NFAT5. Int. Immunol..

[17] Zeng Y., Wang Y., Wu Z., Kang K., Peng X., Peng W., Qu J., Liu L., Raj J.U., Gou D. (2015). MicroRNA-9 enhances the transactivation of nuclear factor of activated T cells by targeting KPNB1 and DYRK1B. Am. J. Physiol. Cell Physiol..

[18] Lin Z., Murtaza I., Wang K., Jiao J., Gao J., Li P.-F. (2009). miR-23a functions downstream of NFATc3 to regulate cardiac hypertrophy. Proc. Natl. Acad. Sci. USA.

[19] Ucar A., Gupta S.K., Fiedler J., Erikci E., Kardasinski M., Batkai S., Dangwal S., Kumarswamy R., Bang C., Holzmann A. (2012). The miRNA-212/132 family regulates both cardiac hypertrophy and cardiomyocyte autophagy. Nat. Commun..

[20] Li Q., Lin X., Yang X., Chang J. (2010). NFATc4 is negatively regulated in miR-133a-mediated cardiomyocyte hypertrophic repression. Am. J. Physiol. Heart Circ. Physiol..

[21] Yoo A.S., Greenwald I. (2005). LIN-12/Notch activation leads to microRNA-mediated down-regulation of Vav in *C. elegans*. Science.

[22] Cook I.H., Evans J., Maldonado-Pérez D., Critchley H.O., Sales K.J., Jabbour H.N. (2009). Prokineticin-1 (PROK1) modulates interleukin (IL)-11 expression via prokineticin receptor 1 (PROKR1) and the calcineurin/NFAT signalling pathway. Mol. Hum. Reprod..

[23] Song D.W., Ryu J.Y., Kim J.O., Kwon E.J., Kim D.H. (2014). The miR-19a / b family positively regulates cardiomyocyte hypertrophy by targeting atrogin-1 and MuRF-1. Biochem. J..

[24] Dirkx E., Gladka M.M., Philippen L.E., Armand A., Kinet V., Leptidis S., Azzouzi H., Salic K., Bourajjaj M., Silva G.J.J. (2013). De Nfat and miR-25 cooperate to reactivate the transcription factor Hand2 in heart failure. Nat. Cell Biol..

[25] Da Costa Martins P.A., Salic K., Gladka M.M., Armand A.-S., Leptidis S., el Azzouzi H., Hansen A., Coenen-de Roo C.J., Bierhuizen M.F., van der Nagel R. (2010). MicroRNA-199b targets the nuclear kinase Dyrk1a in an auto-amplification loop promoting calcineurin/NFAT signalling. Nat. Cell Biol..

[26] Baek D., Villén J., Shin C., Camargo F.D., Gygi S.P., Bartel D.P. (2008). The impact of microRNAs on protein output. Nature.

[27] Betel D., Wilson M., Gabow A., Marks D.S., Sander C. (2008). The microRNA.org resource: Targets and expression. Nucleic Acids Res..

[28] Hsu S.-D., Lin F.-M., Wu W.-Y., Liang C., Huang W.-C., Chan W.-L., Tsai W.-T., Chen G.-Z., Lee C.-J., Chiu C.-M. (2011). miRTarBase: A database curates experimentally validated microRNA–target interactions. Nucleic Acids Res..

[29] Lewis B.P., Burge C.B., Bartel D.P. (2005). Conserved seed pairing, often flanked by adenosines, indicates that thousands of human genes are microRNA targets. Cell.

[30] Yu S., Kim J., Min H., Yoon S. (2014). Ensemble Learning can Significantly Improve Human microRNA Target Prediction. Methods.

[31] Ahmadi H., Ahmadi A., Azimzadeh-Jamalkandi S., Shoorehdeli M.A., Salehzadeh-Yazdi A., Bidkhori G., Masoudi-Nejad A. (2013). HomoTarget: A new algorithm for prediction of microRNA targets in Homo sapiens. Genomics.

[32] Barski A., Jothi R., Cuddapah S., Cui K., Roh T.-Y., Schones D.E., Zhao K. (2009). Chromatin poises miRNA- and protein-coding genes for expression. Genome Res..

[33] Clipstone N.A., Crabtree G.R. (1992). Identification of calcineurin as a key signaling enzyme in lymphocyte-t activation. Nature.

[34] Huang G.N., Huso D.L., Bouyain S., Tu J., McCorkell K.A., May M.J., Zhu Y., Lutz M., Collins S., Dehoff M. (2008). NFAT binding and regulation of T cell activation by the cytoplasmic scaffolding Homer proteins. Science.

[35] Liu Q., Busby J.C., Molkentin J.D. (2009). Interaction between TAK1-TAB1-TAB2 and RCAN1-calcineurin defines a signalling nodal control point. Nat. Cell Biol..

[36] Jang H., Cho E.-J., Youn H.-D. (2007). A new calcineurin inhibition domain in Cabin1. Biochem. Biophys. Res. Commun..

[37] Perrino B.A., Martin B.A. (2001). Ca(2+)- and myristoylation-dependent association of calcineurin with phosphatidylserine. J. Biochem..

[38] Crabtree G.R., Schreiber S.L. (2010). NIH Public Access.

[39] Yang J.H., Li J.H., Shao P., Zhou H., Chen Y.Q., Qu L.H. (2011). StarBase: A database for exploring microRNA-mRNA interaction maps from Argonaute CLIP-Seq and Degradome-Seq data. Nucleic Acids Res..

[40] starBase. http://starbase.sysu.edu.cn/index.php.

[41] Agarwal V., Bell G.W., Nam J.W., Bartel D.P. (2015). Predicting effective microRNA target sites in mammalian mRNAs. Elife.

[42] TargetScanHuman. http://www.targetscan.org.

[43] Wong N., Wang X. (2015). miRDB: An online resource for microRNA target prediction and functional annotations. Nucleic Acids Res..

[44] miRDB. http://mirdb.org/miRDB/index.html.

[45] Vlachos I.S., Paraskevopoulou M.D., Karagkouni D., Georgakilas G., Vergoulis T., Kanellos I., Anastasopoulos I.L., Maniou S., Karathanou K., Kalfakakou D. (2015). DIANA-TarBase v7.0: Indexing more than half a million experimentally supported miRNA:mRNA interactions. Nucleic Acids Res..

[46] TarBase. http://diana.imis.athena-innovation.gr/DianaTools/index.php?r=tarbase/index.

[47] Maragkakis M., Vergoulis T., Alexiou P., Reczko M., Plomaritou K., Gousis M., Kourtis K., Koziris N., Dalamagas T., Hatzigeorgiou A.G. (2011). DIANA-microT Web server upgrade supports Fly and Worm miRNA target prediction and bibliographic miRNA to disease association. Nucleic Acids Res..

[48] DIANA. http://diana.imis.athena-innovation.gr/DianaTools/index.php.

[49] Hsu S.D., Chu C.H., Tsou A.P., Chen S.J., Chen H.C., Hsu P.W.C., Wong Y.H., Chen Y.H., Chen G.H., Huang H. (2008). Da miRNAMap 2.0: Genomic maps of microRNAs in metazoan genomes. Nucleic Acids Res..

[50] miRNAMap. http://mirnamap.mbc.nctu.edu.tw/index.php.

[51] Min H., Yoon S. (2010). Got target?: Computational methods for microRNA target prediction and their extension. Exp. Mol. Med..

[52] Shaw J.P., Utz P.J., Durand D.B., Toole J.J., Emmel E.A., Crabtree G.R. (1988). Identification of a putative regulator of early T cell activation genes. Science.

[53] Baek K.-H., Zaslavsky A., Lynch R.C., Britt C., Okada Y., Siarey R.J., Lensch M.W., Park I.-H., Yoon S.S., Minami T. (2009). Down’s syndrome suppression of tumour growth and the role of the calcineurin inhibitor DSCR1. Nature.

[54] Beurel E., Michalek S.M., Jope R.S. (2010). Innate and adaptive immune responses regulated by glycogen synthase kinase-3 (GSK3). Trends Immunol.

[55] Becker W., Sippl W. (2011). Activation, regulation, and inhibition of DYRK1A. FEBS J..

[56] Schorge S., van de Leemput J., Singleton A., Houlden H., Hardy J. (2010). Human ataxias: A genetic dissection of inositol triphosphate receptor (ITPR1)-dependent signaling. Trends Neurosci..

[57] Sharma S., Findlay G.M., Bandukwala H.S., Oberdoerffer S., Baust B., Li Z., Schmidt V., Hogan P.G., Sacks D.B., Rao A. (2011). Dephosphorylation of the nuclear factor of activated T cells (NFAT) transcription factor is regulated by an RNA-protein scaffold complex. Proc. Natl. Acad. Sci. USA.

[58] Liu Z., Lee J., Krummey S., Lu W., Cai H., Lenardo M.J. (2011). The kinase LRRK2 is a regulator of the transcription factor NFAT that modulates the severity of inflammatory bowel disease. Nat. Immunol..

[59] Li H., Stein A., Dell’Acqua M.L., Pink M.D., Murphy J.G., Hogan P.G. (2012). Balanced interactions of calcineurin with AKAP79 regulate Ca2+–calcineurin–NFAT signaling. Nat. Struct. Mol. Biol..

[60] Hou X., Chen J., Luo Y., Liu F., Xu G., Gao Y. (2013). Silencing of STIM1 attenuates hypoxia-induced PASMCs proliferation via inhibition of the SOC/Ca2+/NFAT pathway. Respir. Res..

[61] Srikanth S., Gwack Y. (2013). Orai1-NFAT signalling pathway triggered by T cell receptor stimulation. Mol. Cells.

[62] miRTarBase. http://mirtarbase.mbc.nctu.edu.tw/index.php.

[63] miRmine. http://guanlab.ccmb.med.umich.edu/mirmine.

[64] Lee Y., Jeon K., Lee J.-T., Kim S., Kim V.N. (2002). MicroRNA maturation: Stepwise processing and subcellular localization. EMBO J..

[65] Lee Y., Kim M., Han J., Yeom K.-H., Lee S., Baek S.H., Kim V.N. (2004). MicroRNA genes are transcribed by RNA polymerase II. EMBO J..

[66] Messeguer X., Escudero R., Farré D., Núñez O., Martínez J., Albà M.M. (2002). PROMO: Detection of known transcription regulatory elements using species-tailored searches. Bioinformatics.

[67] Yang J.H., Li J.H., Jiang S., Zhou H., Qu L.H. (2013). ChIPBase: A database for decoding the transcriptional regulation of long non-coding RNA and microRNA genes from ChIP-Seq data. Nucleic Acids Res..

[68] ChiPBase. http://deepbase.sysu.edu.cn/chipbase/index.php.

[69] Kent W.J., Sugnet C.W., Furey T.S., Roskin K.M., Pringle T.H., Zahler A.M., Haussler D. (2002). The Human Genome Browser at UCSC. Genome Res..

[70] UCSC Genome Bioinformatics. http://genome.ucsc.edu/index.html.

[71] TFSEARCH. http://diyhpl.us/~bryan/irc/protocol-online/protocol-cache/TFSEARCH.html.

[72] PROMO. http://alggen.lsi.upc.es/cgi-bin/promo_v3/promo/promoinit.cgi?dirDB=TF_8.3.

[73] Becskei A., Serrano L. (2000). Engineering stability in gene networks by autoregulation. Nature.

[74] Harris S.L., Levine A.J. (2005). The p53 pathway: Positive and negative feedback loops. Oncogene.

[75] Rosenfeld N., Elowitz M.B., Alon U. (2002). Negative autoregulation speeds the response times of transcription networks. J. Mol. Biol..

[76] Kumar M., Sahu S.K., Kumar R., Subuddhi A., Maji R.K., Jana K., Gupta P., Raffetseder J., Lerm M., Ghosh Z. (2015). MicroRNA let-7 modulates the immune response to mycobacterium tuberculosis infection via control of A20, an inhibitor of the NF-κB pathway. Cell Host Microbe.

[77] Jacob F., Monod J. (1961). Genetic regulatory mechanisms in the synthesis of proteins. J. Mol. Biol..

[78] Helwak A., Kudla G., Dudnakova T., Tollervey D. (2013). Mapping the human miRNA interactome by CLASH reveals frequent noncanonical binding. Cell.

[79] Rey-Giraud F., Hafner M., Ries C.H. (2012). In vitro generation of monocyte-derived macrophages under serum-free conditions improves their tumor promoting functions. PLoS ONE.

[80] Tsitsiou E., Lindsay M.A. (2009). microRNAs and the immune response. Curr. Opin. Pharmacol..

[81] Bazzoni F., Rossato M., Fabbri M., Gaudiosi D., Mirolo M., Mori L., Tamassia N., Mantovani A., Cassatella M.A., Locati M. (2009). Induction and regulatory function of miR-9 in human monocytes and neutrophils exposed to proinflammatory signals. Proc. Natl. Acad. Sci. USA.

[82] Thai T.-H., Calado D.P., Casola S., Ansel K.M., Xiao C., Xue Y., Murphy A., Frendewey D., Valenzuela D., Kutok J.L. (2007). Regulation of the germinal center response by microRNA-155. Science.

[83] Rothenberg Melissa K., Mingler M.E., Cole E.T., Orkin S.H., Thomas X., Lu B.J., Hartner J., Lim E.-J., Fabry V., Lu T.X. (2011). Delayed-Type Hypersensitivity MicroRNA-21 Limits *in Vivo* Immune Response-Mediated Activation of the IL-12/IFN-g Pathway, Th1 Polarization, and the Severity of Delayed-Type Hypersensitivity. J. Immunol..

[84] Gabriely G., Wurdinger T., Kesari S., Esau C.C., Burchard J., Linsley P.S., Krichevsky A.M. (2008). MicroRNA 21 promotes glioma invasion by targeting matrix metalloproteinase regulators. Mol. Cell. Biol..

[85] Liu G., Min H., Yue S., Chen C.Z. (2008). Pre-miRNA loop nucleotides control the distinct activities of mir-181a-1 and mir-181c in early T cell development. PLoS ONE.

[86] Kishore S., Jaskiewicz L., Burger L., Hausser J., Khorshid M., Zavolan M. (2011). A quantitative analysis of CLIP methods for identifying binding sites of RNA-binding proteins. Nat. Methods.

